# Supplementary figures and images for: A single cysteine residue in vimentin regulates long non-coding RNA XIST to suppress epithelial–mesenchymal transition and stemness in breast cancer
Source: eLife. 2025 Jul 21;14:RP104191. doi: 10.7554/eLife.104191 (PMC12279371; doi:10.7554/eLife.104191)

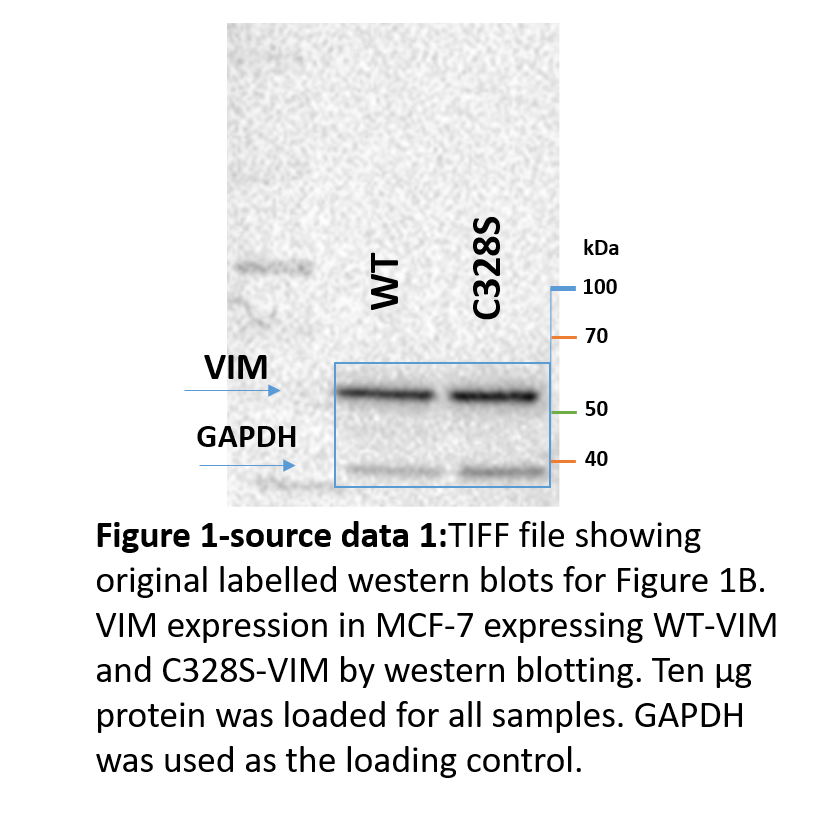

Supplement: Figure 1—source data 1. [file elife-104191-fig1-data1.zip › Figure 1-source data 1.tif]

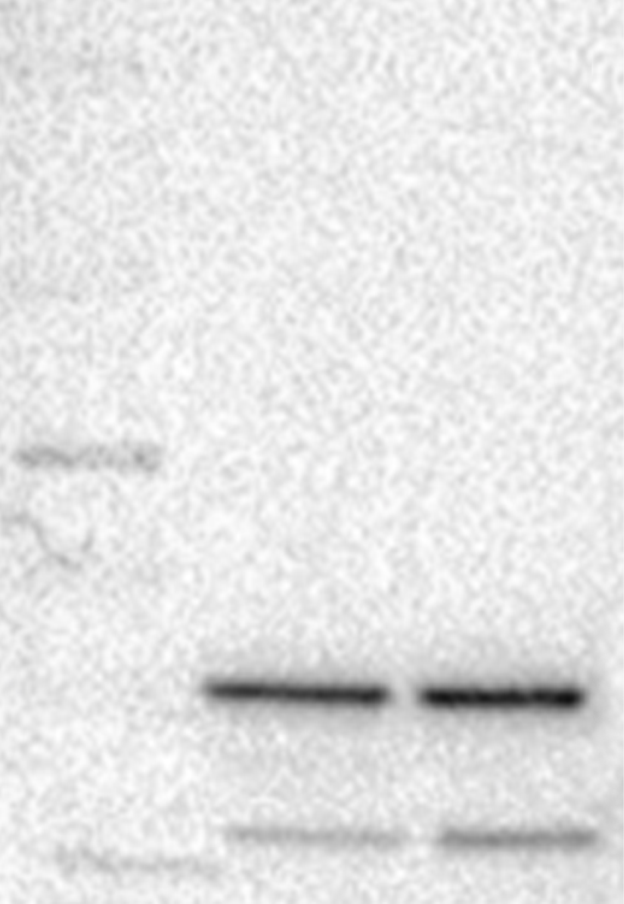

Supplement: Figure 1—source data 2. [file elife-104191-fig1-data2.zip › Figure 1C source data-2.tif]

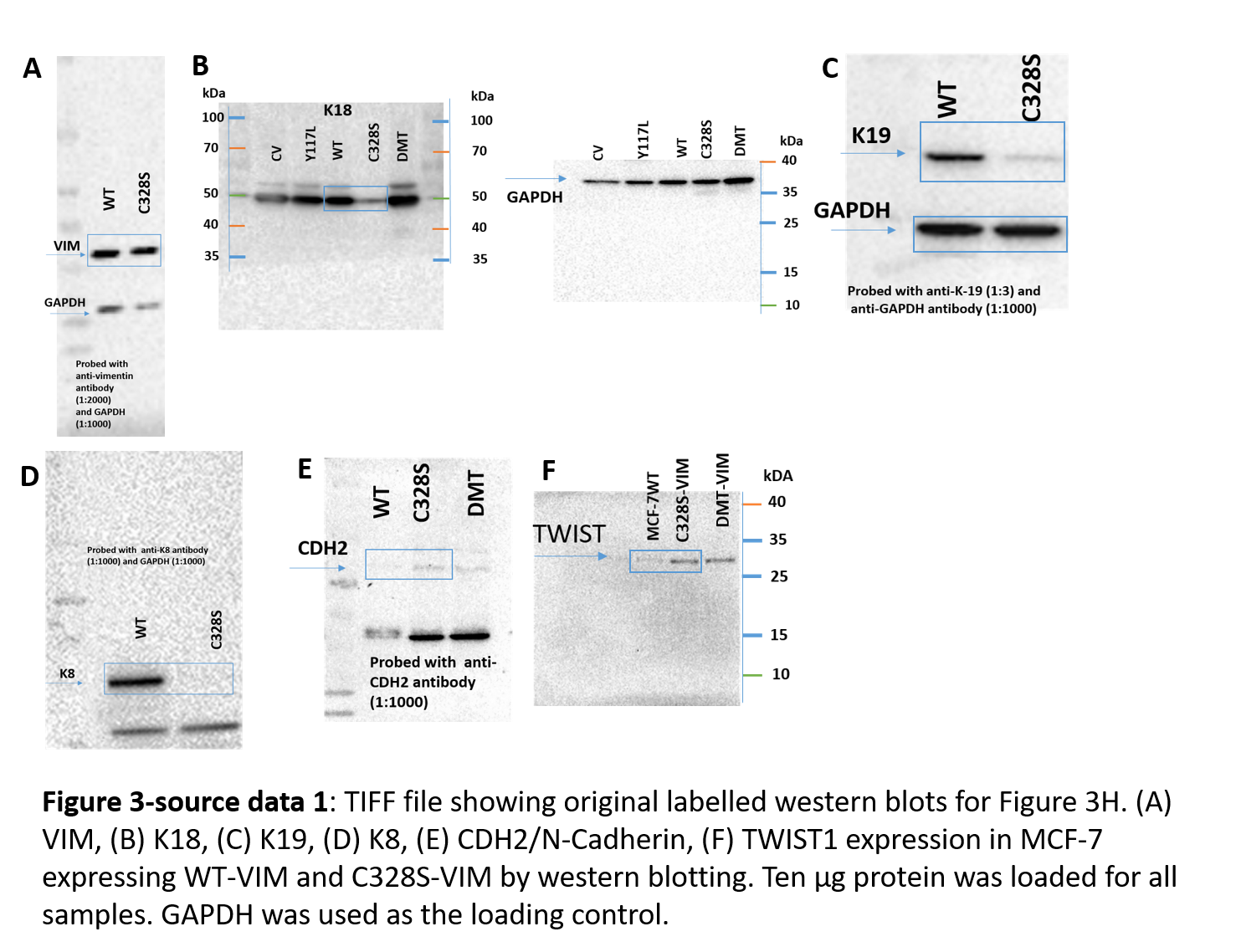

Supplement: Figure 3—source data 1. [file elife-104191-fig3-data1.zip › Figure 3 source data 1.tif]

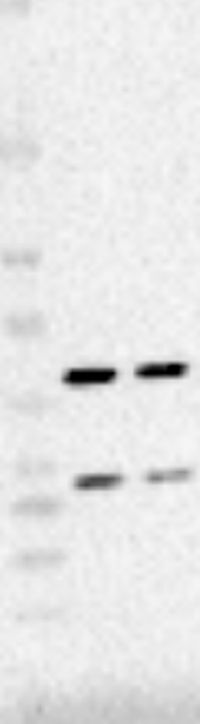

Supplement: Figure 3—source data 2. [file elife-104191-fig3-data2.zip › VIM.tif]

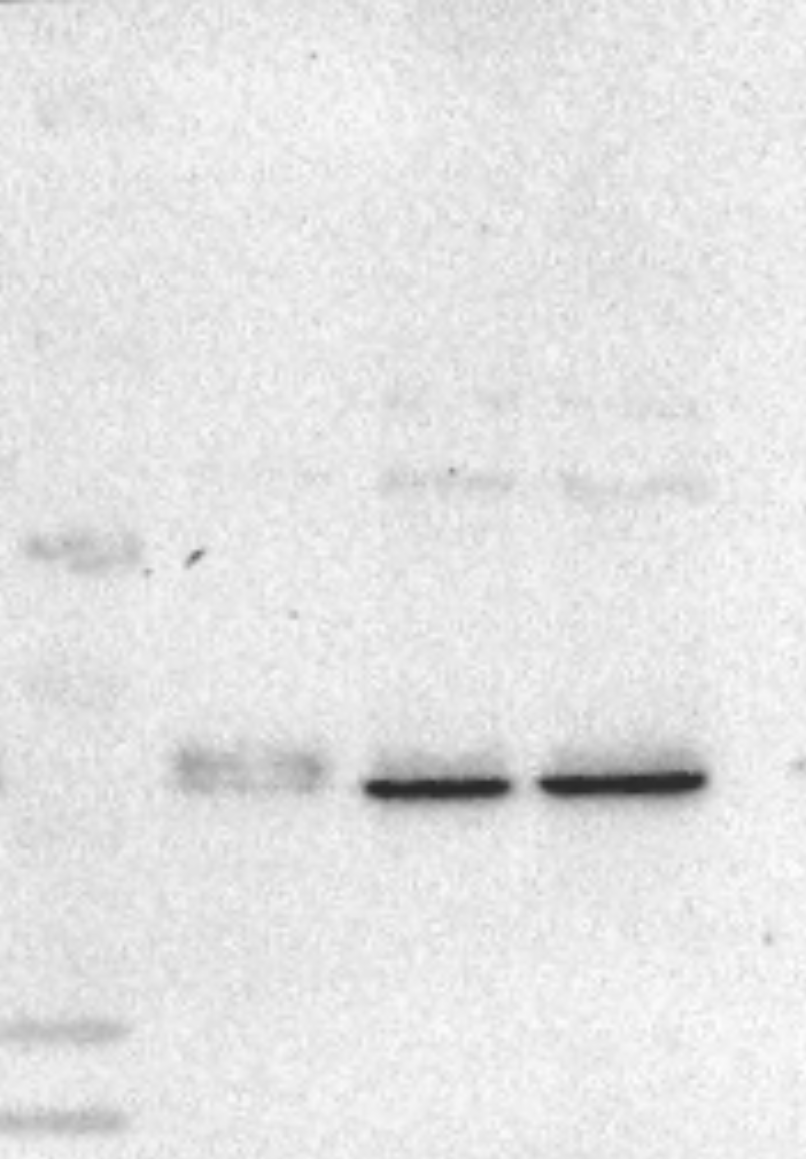

Supplement: Figure 3—source data 2. [file elife-104191-fig3-data2.zip › CDH2.tif]

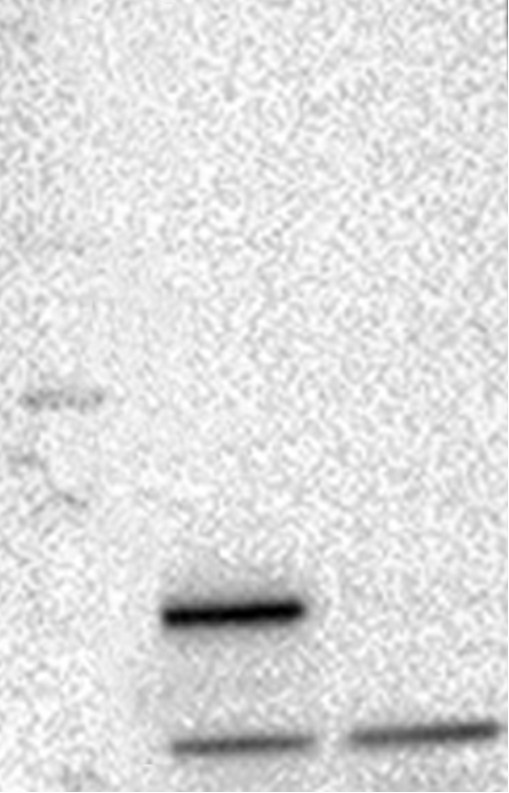

Supplement: Figure 3—source data 2. [file elife-104191-fig3-data2.zip › K8.tif]

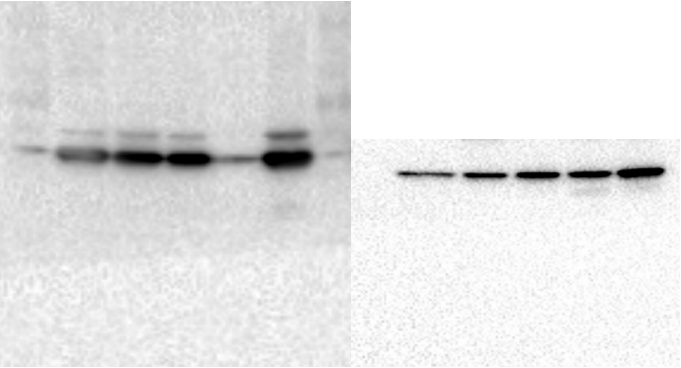

Supplement: Figure 3—source data 2. [file elife-104191-fig3-data2.zip › K18.tif]

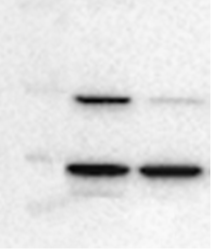

Supplement: Figure 3—source data 2. [file elife-104191-fig3-data2.zip › K19.tif]

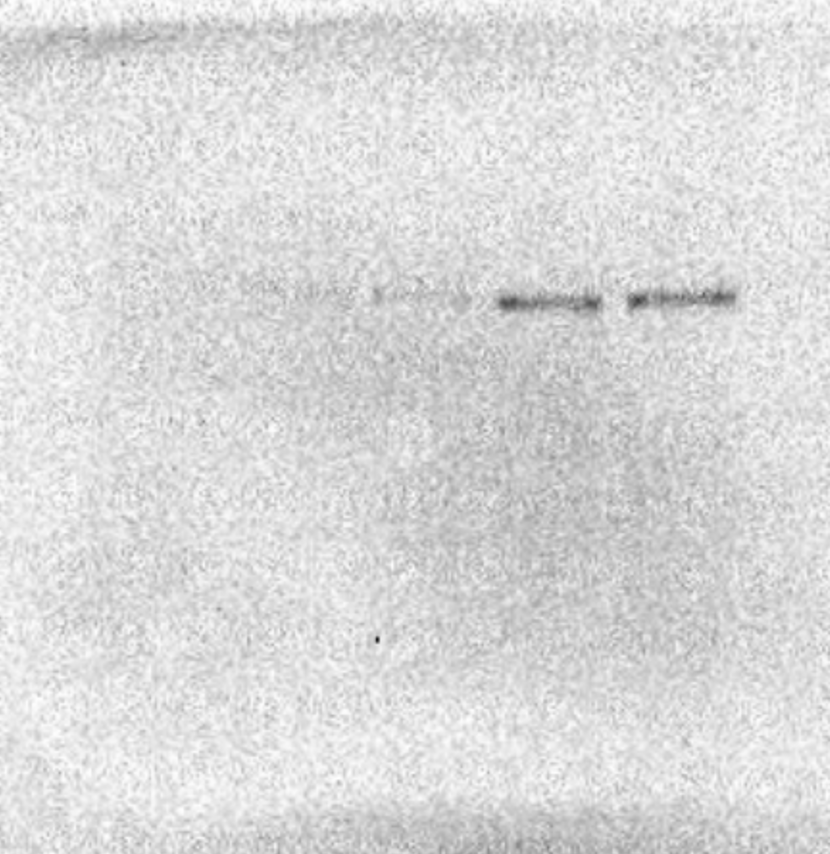

Supplement: Figure 3—source data 2. [file elife-104191-fig3-data2.zip › Twist.tif]

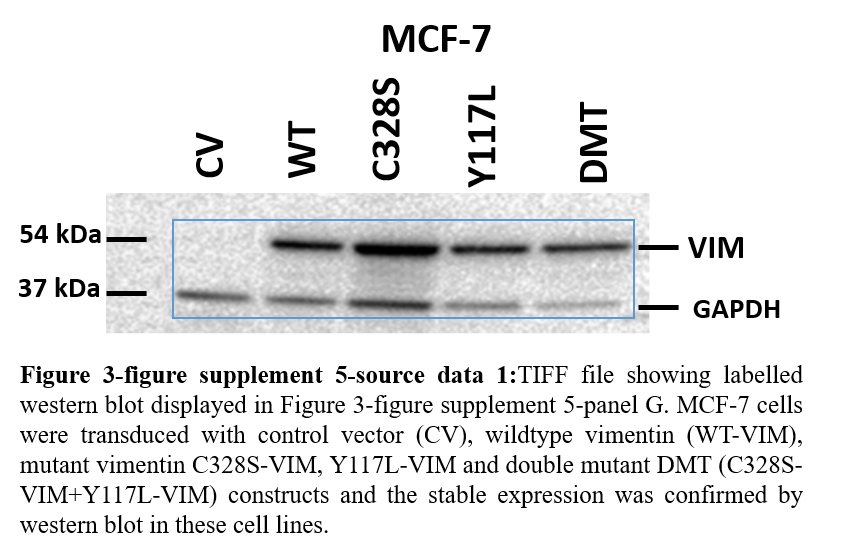

Supplement: Figure 3—figure supplement 5—source data 1. [file elife-104191-fig3-figsupp5-data1.zip › Figure 3-figure supplement 5-source data-1.tif]

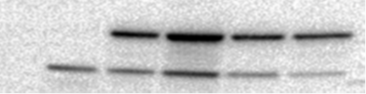

Supplement: Figure 3—figure supplement 5—source data 2. [file elife-104191-fig3-figsupp5-data2.zip › Figure 3- figure supplement 5-source data-2.tif]

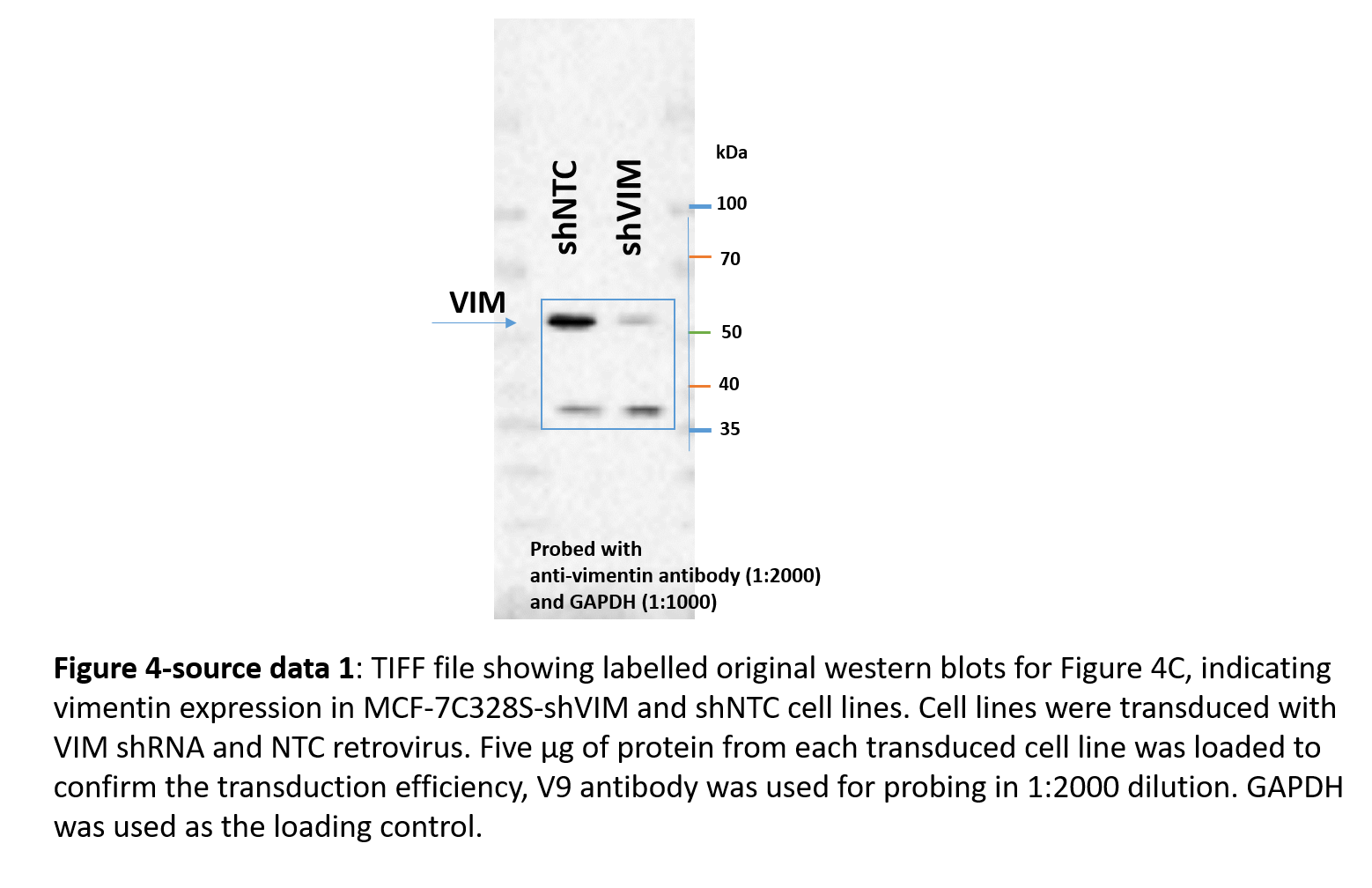

Supplement: Figure 4—source data 1. [file elife-104191-fig4-data1.zip › Figure 4-source data 1.tif]

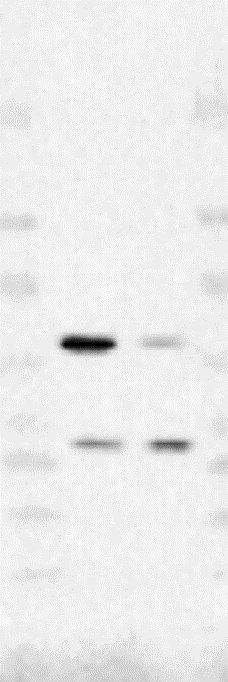

Supplement: Figure 4—source data 2. [file elife-104191-fig4-data2.zip › Figure 4C source data-2.tif]
