# Supplementary material for: A single cysteine residue in vimentin regulates long non-coding RNA XIST to suppress epithelial–mesenchymal transition and stemness in breast cancer
Source: eLife. 2025 Jul 21;14:RP104191. doi: 10.7554/eLife.104191 (PMC12279371; doi:10.7554/eLife.104191)
Supplement: Supplementary file 1. [file elife-104191-supp1.docx]

**Supplementary File 1: List of upregulated DEGs (cut off padj=0.00009).**

| Gene_name | Gene ID | log2Fold Change | padj |
| --- | --- | --- | --- |
| *XIST* | ENSG00000229807 | 11.79645 | 1.96E-93 |
| *IGF2BP1* | ENSG00000159217 | 8.245026 | 1.30E-50 |
| *FSTL1* | ENSG00000163430 | 8.139257 | 2.61E-40 |
| *CNN3* | ENSG00000117519 | 10.10871 | 1.46E-39 |
| *AKAP12* | ENSG00000131016 | 7.116653 | 3.28E-35 |
| *HMGA2* | ENSG00000149948 | 8.620275 | 3.35E-34 |
| *ADGRL2* | ENSG00000117114 | 6.591145 | 2.79E-32 |
| *Septin 6* | ENSG00000125354 | 7.320673 | 3.65E-32 |
| *CDH2* | ENSG00000170558 | 7.722077 | 9.49E-31 |
| *JAM3* | ENSG00000166086 | 6.779312 | 1.04E-29 |
| *COL4A2* | ENSG00000134871 | 7.216354 | 2.60E-29 |
| *CDKN2A* | ENSG00000147889 | 5.749695 | 2.15E-27 |
| *FLNC* | ENSG00000128591 | 8.10231 | 2.38E-27 |
| *RAB34* | ENSG00000109113 | 8.462863 | 2.76E-27 |
| *MAPRE2* | ENSG00000166974 | 5.772006 | 1.22E-26 |
| *HOXB9* | ENSG00000170689 | 5.695711 | 5.44E-26 |
| *SMOC1* | ENSG00000198732 | 6.677228 | 7.77E-26 |
| *NCAM1* | ENSG00000149294 | 6.737192 | 7.79E-26 |
| *FBN2* | ENSG00000138829 | 5.50309 | 6.04E-24 |
| *ALDH2* | ENSG00000111275 | 8.003519 | 1.01E-23 |
| *LAMC3* | ENSG00000050555 | 5.986133 | 1.31E-21 |
| *CCND2* | ENSG00000118971 | 9.643044 | 7.78E-21 |
| *ANK2* | ENSG00000145362 | 8.760965 | 9.82E-21 |
| *ZEB1* | ENSG00000148516 | 7.687737 | 1.04E-20 |
| *ADAM23* | ENSG00000114948 | 6.225849 | 1.04E-20 |
| *HOXA10* | ENSG00000253293 | 6.876391 | 1.58E-20 |
| *HOXA5* | ENSG00000106004 | 5.081385 | 1.64E-20 |
| *MARK1* | ENSG00000116141 | 5.651885 | 1.66E-20 |
| *ADAMTS3* | ENSG00000156140 | 7.747103 | 4.02E-20 |
| *TCF4* | ENSG00000196628 | 9.505749 | 4.43E-20 |
| *HOXB13* | ENSG00000159184 | 5.58403 | 5.70E-20 |
| *FOXP2* | ENSG00000128573 | 8.044315 | 6.79E-20 |
| *GDF7* | ENSG00000143869 | 6.120914 | 8.16E-20 |
| *LINC02381* | ENSG00000250742 | 6.953446 | 1.78E-19 |
| *PURPL* | ENSG00000250337 | 6.328863 | 2.34E-19 |
| *HOXA3* | ENSG00000105997 | 7.224113 | 3.14E-19 |
| *HOXA11* | ENSG00000005073 | 7.199001 | 4.01E-19 |
| *KIF5A* | ENSG00000155980 | 6.3359 | 4.35E-19 |
| *ITGA1* | ENSG00000213949 | 6.752507 | 9.82E-19 |
| *PLAT* | ENSG00000104368 | 8.445353 | 2.55E-18 |
| *COL4A6* | ENSG00000197565 | 9.021868 | 3.63E-18 |
| *BARX1* | ENSG00000131668 | 10.43802 | 4.06E-18 |
| *NECTIN3* | ENSG00000177707 | 4.969662 | 4.82E-18 |
| *TCF19* | ENSG00000137310 | 5.805573 | 8.18E-18 |
| *LAMB1* | ENSG00000091136 | 4.93954 | 1.32E-17 |
| *CAPN2* | ENSG00000162909 | 4.383822 | 4.43E-17 |
| *ARHGAP22* | ENSG00000128805 | 6.615097 | 5.34E-17 |
| *IGF2BP2* | ENSG00000073792 | 4.670273 | 5.44E-17 |
| *CIB2* | ENSG00000136425 | 5.567477 | 8.61E-17 |
| *ALX4* | ENSG00000052850 | 5.715407 | 8.88E-17 |
| *SNAI2* | ENSG00000019549 | 4.392217 | 1.02E-16 |
| *FMN2* | ENSG00000155816 | 6.063439 | 5.60E-16 |
| *HOTAIRM1* | ENSG00000233429 | 5.231698 | 6.98E-16 |
| *IRF8* | ENSG00000140968 | 6.063818 | 8.63E-16 |
| *COL14A1* | ENSG00000187955 | 5.501122 | 9.83E-16 |
| *WNT5A* | ENSG00000114251 | 4.462759 | 1.74E-15 |
| *HOXA6* | ENSG00000106006 | 6.027171 | 3.08E-15 |
| *IGF2BP3* | ENSG00000136231 | 12.82133 | 3.22E-15 |
| *HLA-B* | ENSG00000234745 | 12.77643 | 4.15E-15 |
| *ADGRL3* | ENSG00000150471 | 5.66715 | 6.18E-15 |
| *INA* | ENSG00000148798 | 12.64546 | 8.20E-15 |
| *C17orf51* | ENSG00000212719 | 12.56534 | 1.26E-14 |
| *PCOLCE2* | ENSG00000163710 | 4.396516 | 1.29E-14 |
| *TFCP2* | ENSG00000135457 | 12.55276 | 1.33E-14 |
| *LAMA1* | ENSG00000101680 | 12.53328 | 1.48E-14 |
| *MIR222HG* | ENSG00000270069 | 5.99406 | 1.56E-14 |
| *COL4A1* | ENSG00000187498 | 12.09111 | 1.71E-14 |
| *MXRA7* | ENSG00000182534 | 4.39984 | 2.28E-14 |
| *DSC3* | ENSG00000134762 | 11.93573 | 3.91E-14 |
| *RAB39A* | ENSG00000179331 | 6.873654 | 4.14E-14 |
| *BCL6B* | ENSG00000161940 | 8.01962 | 5.02E-14 |
| *TP73-AS1* | ENSG00000227372 | 12.29649 | 5.05E-14 |
| *FYN* | ENSG00000010810 | 3.989116 | 6.24E-14 |
| *FLT1* | ENSG00000102755 | 8.037025 | 9.81E-14 |
| *RARB* | ENSG00000077092 | 6.371803 | 1.06E-13 |
| *HOXA9* | ENSG00000078399 | 11.71772 | 1.22E-13 |
| *FOXF2* | ENSG00000137273 | 12.05026 | 1.80E-13 |
| *GSPT2* | ENSG00000189369 | 11.84337 | 5.18E-13 |
| *CASP10* | ENSG00000003400 | 5.502836 | 5.56E-13 |
| *ITGA4* | ENSG00000115232 | 11.69636 | 1.08E-12 |
| *ZEB2* | ENSG00000169554 | 11.64134 | 1.43E-12 |
| *PCDH7* | ENSG00000169851 | 4.022888 | 1.45E-12 |
| *CAMK4* | ENSG00000152495 | 11.60755 | 1.67E-12 |
| *HOXA1* | ENSG00000105991 | 8.702472 | 1.93E-12 |
| *MAP1B* | ENSG00000131711 | 3.759399 | 2.30E-12 |
| *HOXA13* | ENSG00000106031 | 4.546559 | 2.34E-12 |
| *HOXB3* | ENSG00000120093 | 4.563872 | 2.86E-12 |
| *COL25A1* | ENSG00000188517 | 11.48404 | 3.13E-12 |
| *ARPIN* | ENSG00000242498 | 3.758205 | 4.05E-12 |
| *NEXN* | ENSG00000162614 | 6.865973 | 5.42E-12 |
| *TWIST1* | ENSG00000122691 | 8.467432 | 8.16E-12 |
| *ARHGEF6* | ENSG00000129675 | 4.102098 | 8.22E-12 |
| *FOXG1* | ENSG00000176165 | 10.85672 | 9.16E-12 |
| *CDK14* | ENSG00000058091 | 4.146313 | 1.11E-11 |
| *BCL11A* | ENSG00000119866 | 11.21047 | 1.17E-11 |
| *CDH12* | ENSG00000154162 | 11.0138 | 2.99E-11 |
| *COL26A1* | ENSG00000160963 | 6.314866 | 3.26E-11 |
| *FGF2* | ENSG00000138685 | 10.98633 | 3.39E-11 |
| *CNTN1* | ENSG00000018236 | 10.96843 | 3.71E-11 |
| *ADGRB3* | ENSG00000135298 | 7.294488 | 5.22E-11 |
| *AFAP1L1* | ENSG00000157510 | 5.212391 | 6.18E-11 |
| *LAMA4* | ENSG00000112769 | 10.73255 | 1.11E-10 |
| *HOXB4* | ENSG00000182742 | 5.511675 | 1.22E-10 |
| *TWIST2* | ENSG00000233608 | 10.7037 | 1.29E-10 |
| *FNDC1* | ENSG00000164694 | 10.65256 | 1.66E-10 |
| *DOCK3* | ENSG00000088538 | 3.89674 | 1.95E-10 |
| *COL13A1* | ENSG00000197467 | 6.383551 | 2.78E-10 |
| *SOX8* | ENSG00000005513 | 5.255238 | 3.34E-10 |
| *PCDHGA11* | ENSG00000253873 | 4.901264 | 3.81E-10 |
| *ACTN2* | ENSG00000077522 | 7.732494 | 1.08E-09 |
| *SOX6* | ENSG00000110693 | 4.518772 | 1.32E-09 |
| *MAP2K6* | ENSG00000108984 | 3.709073 | 1.63E-09 |
| *PRKCQ* | ENSG00000065675 | 10.14948 | 1.66E-09 |
| *TNFRSF10D* | ENSG00000173530 | 9.71865 | 1.78E-09 |
| *CDH23* | ENSG00000107736 | 4.402438 | 1.95E-09 |
| *COL4A5* | ENSG00000188153 | 3.48696 | 2.16E-09 |
| *CDHR1* | ENSG00000148600 | 9.651466 | 2.21E-09 |
| *PCDH9* | ENSG00000184226 | 3.418713 | 2.38E-09 |
| *PCDH18* | ENSG00000189184 | 9.91912 | 4.70E-09 |
| *COL5A2* | ENSG00000204262 | 3.35551 | 1.45E-08 |
| *ITGA8* | ENSG00000077943 | 9.168304 | 1.30E-07 |
| *LBR* | ENSG00000143815 | 3.593871 | 1.43E-07 |
| *CD40* | ENSG00000101017 | 8.980084 | 2.84E-07 |
| *CDC7* | ENSG00000097046 | 3.559382 | 5.89E-07 |
| *SMAD9* | ENSG00000120693 | 2.784389 | 7.88E-07 |
| *ARHGAP31* | ENSG00000031081 | 3.096402 | 8.21E-07 |
| *MMP2* | ENSG00000087245 | 8.727277 | 8.90E-07 |
| *SKP2* | ENSG00000145604 | 2.771911 | 9.88E-07 |
| *EGFLAM* | ENSG00000164318 | 8.663549 | 1.10E-06 |
| *ADAMTS10* | ENSG00000142303 | 4.201208 | 1.23E-06 |
| *CD109* | ENSG00000156535 | 3.27812 | 1.82E-06 |
| *RASGRF2* | ENSG00000113319 | 3.61194 | 1.87E-06 |
| *COL2A1* | ENSG00000139219 | 3.080835 | 2.28E-06 |
| *MPP6* | ENSG00000105926 | 2.993963 | 2.28E-06 |
| *PINCR* | ENSG00000224294 | 8.499739 | 2.31E-06 |
| *CD70* | ENSG00000125726 | 4.550187 | 2.44E-06 |
| *KRT222* | ENSG00000213424 | 8.451071 | 2.58E-06 |
| *NRXN2* | ENSG00000110076 | 4.226864 | 2.60E-06 |
| *DNM3* | ENSG00000197959 | 3.346947 | 2.99E-06 |
| *CD19* | ENSG00000177455 | 8.405213 | 3.10E-06 |
| *ITGA9* | ENSG00000144668 | 4.800421 | 3.15E-06 |
| *CNTNAP3B* | ENSG00000154529 | 8.391128 | 3.29E-06 |
| *TCL6* | ENSG00000187621 | 8.383689 | 3.38E-06 |
| *CD83* | ENSG00000112149 | 2.894394 | 3.64E-06 |
| *LZTS1* | ENSG00000061337 | 8.35354 | 3.82E-06 |
| *CDCA7* | ENSG00000144354 | 2.785288 | 3.82E-06 |
| *COL24A1* | ENSG00000171502 | 4.430698 | 3.84E-06 |
| *JCAD* | ENSG00000165757 | 2.713746 | 3.88E-06 |
| *KIFAP3* | ENSG00000075945 | 2.698616 | 4.56E-06 |
| *KIF17* | ENSG00000117245 | 3.216778 | 5.82E-06 |
| *LAMA2* | ENSG00000196569 | 3.716597 | 6.18E-06 |
| *FGF5* | ENSG00000138675 | 8.162665 | 8.32E-06 |
| *AKT3* | ENSG00000117020 | 7.905812 | 8.49E-06 |
| *KIF15* | ENSG00000163808 | 3.136117 | 9.94E-06 |
| *ITGB3BP* | ENSG00000142856 | 3.614646 | 1.01E-05 |
| *PAK1* | ENSG00000149269 | 2.421808 | 1.44E-05 |
| *HOXA7* | ENSG00000122592 | 3.302447 | 1.51E-05 |
| *CDON* | ENSG00000064309 | 2.538878 | 1.73E-05 |
| *AMPH* | ENSG00000078053 | 2.580854 | 1.92E-05 |
| *CEP85L* | ENSG00000111860 | 2.728596 | 2.00E-05 |
| *TNFRSF13C* | ENSG00000159958 | 3.154884 | 2.02E-05 |
| *ANGPT1* | ENSG00000154188 | 7.365613 | 2.20E-05 |
| *DNAH6* | ENSG00000115423 | 6.033898 | 2.53E-05 |
| *FAT3* | ENSG00000165323 | 7.329281 | 2.62E-05 |
| *CDC25A* | ENSG00000164045 | 2.269125 | 2.84E-05 |
| *CCSAP* | ENSG00000154429 | 2.767498 | 3.22E-05 |
| *SOX21* | ENSG00000125285 | 5.969039 | 3.49E-05 |
| *HOXA2* | ENSG00000105996 | 7.227463 | 3.50E-05 |
| *HSF2* | ENSG00000025156 | 2.667201 | 3.85E-05 |
| *HOXA4* | ENSG00000197576 | 3.091659 | 4.62E-05 |
| *FGF17* | ENSG00000158815 | 5.801019 | 4.90E-05 |
| *RAB9B* | ENSG00000123570 | 2.95757 | 5.23E-05 |
| *PRKD1* | ENSG00000184304 | 2.453299 | 5.58E-05 |
| *TLR6* | ENSG00000174130 | 4.521907 | 5.69E-05 |
| *MIR221* | ENSG00000207870 | 7.665559 | 5.76E-05 |
| *PLXNA2* | ENSG00000076356 | 3.033146 | 5.80E-05 |
| *ALDH8A1* | ENSG00000118514 | 4.900099 | 6.10E-05 |
| *MN1* | ENSG00000169184 | 2.6547 | 6.91E-05 |
| *TFPI2* | ENSG00000105825 | 3.185966 | 6.97E-05 |
| *ADGRA2* | ENSG00000020181 | 2.740551 | 7.32E-05 |
| *CEP112* | ENSG00000154240 | 2.829766 | 7.36E-05 |
| *FAT4* | ENSG00000196159 | 2.282895 | 7.50E-05 |
| *TTBK2* | ENSG00000128881 | 2.501309 | 7.53E-05 |
| *PAK3* | ENSG00000077264 | 5.712818 | 7.54E-05 |
| *RASSF2* | ENSG00000101265 | 2.731273 | 8.29E-05 |
