# Supplementary material for: A single cysteine residue in vimentin regulates long non-coding RNA XIST to suppress epithelial–mesenchymal transition and stemness in breast cancer
Source: eLife. 2025 Jul 21;14:RP104191. doi: 10.7554/eLife.104191 (PMC12279371; doi:10.7554/eLife.104191)
Supplement: Supplementary file 2. [file elife-104191-supp2.docx]

**Supplementary File 2: List of downregulated DEGs (cut off padj=0.00009).**

| **Gene Name** | **Gene ID** | **Log2fold Change** | **padj** |
| --- | --- | --- | --- |
| *KRT19* | ENSG00000171345 | -12.5017 | 1.04E-92 |
| *KRT80* | ENSG00000167767 | -13.0018 | 3.65E-75 |
| *KRT8* | ENSG00000170421 | -10.1552 | 5.61E-74 |
| *DSCAM-AS1* | ENSG00000235123 | -11.5496 | 1.76E-62 |
| *S100A16* | ENSG00000188643 | -10.7403 | 8.54E-58 |
| *CD24* | ENSG00000272398 | -8.60434 | 9.68E-58 |
| *CDH1* | ENSG00000039068 | -8.03511 | 1.38E-56 |
| *ESRP2* | ENSG00000103067 | -9.60887 | 2.27E-49 |
| *ESRP1* | ENSG00000104413 | -9.95392 | 2.27E-49 |
| *TACSTD2* | ENSG00000184292 | -11.8701 | 3.22E-48 |
| *ERBB3* | ENSG00000065361 | -8.03517 | 4.43E-48 |
| *L1CAM* | ENSG00000198910 | -9.39655 | 3.12E-47 |
| *WISP2* | ENSG00000064205 | -11.8286 | 8.13E-47 |
| *EPPK1* | ENSG00000261150 | -9.79614 | 1.69E-46 |
| *CLDN3* | ENSG00000165215 | -13.4255 | 1.18E-44 |
| *PKP3* | ENSG00000184363 | -11.4093 | 1.18E-44 |
| *FOXA1* | ENSG00000129514 | -7.20191 | 1.27E-44 |
| *CLDN7* | ENSG00000181885 | -9.11937 | 1.02E-42 |
| *CLDN4* | ENSG00000189143 | -9.19594 | 1.62E-42 |
| *WNT7B* | ENSG00000188064 | -9.18573 | 5.58E-42 |
| *RAB25* | ENSG00000132698 | -9.4677 | 7.90E-42 |
| *PPL* | ENSG00000118898 | -8.41424 | 1.51E-41 |
| *KRT18* | ENSG00000111057 | -7.56932 | 6.86E-41 |
| *KRT81* | ENSG00000205426 | -15.9626 | 4.93E-39 |
| *ADGRG1* | ENSG00000205336 | -11.7431 | 6.99E-39 |
| *CDH3* | ENSG00000062038 | -7.39085 | 1.16E-37 |
| *EPCAM* | ENSG00000119888 | -6.7076 | 7.64E-36 |
| *S100A14* | ENSG00000189334 | -11.3462 | 1.93E-33 |
| *PDGFB* | ENSG00000100311 | -8.66131 | 1.65E-32 |
| *KRT86* | ENSG00000170442 | -9.43315 | 1.21E-31 |
| *ITGB4* | ENSG00000132470 | -7.09122 | 8.61E-27 |
| *NUPR1* | ENSG00000176046 | -6.76598 | 1.42E-26 |
| *BCL3* | ENSG00000069399 | -6.41802 | 5.49E-26 |
| *NECTIN4* | ENSG00000143217 | -6.8834 | 1.33E-25 |
| *PTPN6* | ENSG00000111679 | -6.68543 | 9.79E-25 |
| *KRT8P3* | ENSG00000254285 | -10.1481 | 1.30E-24 |
| *CLDN9* | ENSG00000213937 | -10.5792 | 1.55E-24 |
| *APOD* | ENSG00000189058 | -6.66367 | 3.88E-24 |
| *TNFRSF12A* | ENSG00000006327 | -5.95843 | 2.38E-23 |
| *TGFBI* | ENSG00000120708 | -9.49209 | 3.90E-23 |
| *SHH* | ENSG00000164690 | -6.98255 | 6.98E-23 |
| *PCDHGB5* | ENSG00000276547 | -7.79629 | 7.94E-23 |
| *PARP12* | ENSG00000059378 | -7.62925 | 8.78E-23 |
| *CLDN1* | ENSG00000163347 | -7.09195 | 2.18E-22 |
| *HMCN1* | ENSG00000143341 | -5.09272 | 5.33E-22 |
| *CD44* | ENSG00000026508 | -6.34163 | 1.50E-21 |
| *BCAS3* | ENSG00000141376 | -5.08945 | 5.08E-21 |
| *JUNB* | ENSG00000171223 | -4.9864 | 4.86E-20 |
| *IKBKE* | ENSG00000263528 | -6.87871 | 9.30E-20 |
| *LAMB3* | ENSG00000196878 | -7.44425 | 2.08E-19 |
| *YBX2* | ENSG00000006047 | -5.31419 | 2.94E-19 |
| *KIF12* | ENSG00000136883 | -5.68644 | 4.60E-19 |
| *TGFA* | ENSG00000163235 | -5.3384 | 5.31E-19 |
| *PARD6B* | ENSG00000124171 | -5.10334 | 9.58E-19 |
| *ICAM1* | ENSG00000090339 | -6.44375 | 9.71E-19 |
| *CTNND2* | ENSG00000169862 | -14.3521 | 1.25E-18 |
| *COL3A1* | ENSG00000168542 | -4.46134 | 2.29E-18 |
| *PCDH1* | ENSG00000156453 | -5.71508 | 2.59E-18 |
| *PCDHB2* | ENSG00000112852 | -6.21841 | 3.75E-18 |
| *TFAP2C* | ENSG00000087510 | -4.48661 | 3.81E-18 |
| *S100A9* | ENSG00000163220 | -6.7512 | 4.51E-18 |
| *BOK* | ENSG00000176720 | -5.39943 | 4.90E-18 |
| *CD9* | ENSG00000010278 | -4.54939 | 6.14E-18 |
| *CDC42BPG* | ENSG00000171219 | -5.20928 | 9.57E-18 |
| *FIBCD1* | ENSG00000130720 | -5.56018 | 2.00E-17 |
| *S100A11* | ENSG00000163191 | -4.40166 | 3.41E-17 |
| *HSPB8* | ENSG00000152137 | -4.34555 | 5.70E-17 |
| *AIFM2* | ENSG00000042286 | -5.09404 | 1.77E-16 |
| *PARP9* | ENSG00000138496 | -4.99279 | 2.04E-16 |
| *RHOD* | ENSG00000173156 | -13.3707 | 2.27E-16 |
| *SOX3* | ENSG00000134595 | -6.13532 | 2.59E-16 |
| *RAB26* | ENSG00000167964 | -5.14424 | 2.89E-16 |
| *NCAM2* | ENSG00000154654 | -13.2264 | 5.78E-16 |
| *PARP14* | ENSG00000173193 | -9.96466 | 9.67E-16 |
| *BIK* | ENSG00000100290 | -4.95456 | 1.91E-15 |
| *CDKN1A* | ENSG00000124762 | -4.22775 | 3.01E-15 |
| *RAB17* | ENSG00000124839 | -12.7669 | 3.61E-15 |
| *ITGB6* | ENSG00000115221 | -12.736 | 6.79E-15 |
| *PKP1* | ENSG00000081277 | -12.8297 | 7.34E-15 |
| *DSCAM* | ENSG00000171587 | -12.5465 | 7.91E-15 |
| *PCDHB3* | ENSG00000113205 | -9.39072 | 2.70E-14 |
| *S100A6* | ENSG00000197956 | -12.3847 | 3.88E-14 |
| *JUP* | ENSG00000173801 | -4.7403 | 4.40E-14 |
| *BCAM* | ENSG00000187244 | -4.80314 | 6.64E-14 |
| *PCDHA6* | ENSG00000081842 | -12.2947 | 1.27E-13 |
| *KDF1* | ENSG00000175707 | -12.3892 | 1.45E-13 |
| *PLXNB1* | ENSG00000164050 | -4.63775 | 1.88E-13 |
| *AMIGO2* | ENSG00000139211 | -4.19602 | 3.07E-13 |
| *ITGA3* | ENSG00000005884 | -4.4519 | 8.26E-13 |
| *FMN1* | ENSG00000248905 | -6.74342 | 1.27E-12 |
| *MAPK11* | ENSG00000185386 | -4.64408 | 1.34E-12 |
| *PARD6A* | ENSG00000102981 | -5.05535 | 1.39E-12 |
| *RAB27B* | ENSG00000041353 | -5.19445 | 2.15E-12 |
| *KRTCAP3* | ENSG00000157992 | -4.809 | 2.66E-12 |
| *SDC4* | ENSG00000124145 | -3.55913 | 6.44E-12 |
| *DRAM1* | ENSG00000136048 | -3.91881 | 8.11E-12 |
| *PCDHA11* | ENSG00000249158 | -11.3521 | 8.42E-12 |
| *PCDHGA2* | ENSG00000081853 | -11.2075 | 1.70E-11 |
| *KRT7* | ENSG00000135480 | -11.1068 | 1.85E-11 |
| *KRT15* | ENSG00000171346 | -11.2416 | 1.95E-11 |
| *FGF13* | ENSG00000129682 | -5.26285 | 2.46E-11 |
| *TGFB2* | ENSG00000092969 | -4.75549 | 3.11E-11 |
| *BMF* | ENSG00000104081 | -3.66168 | 3.35E-11 |
| *NFKBIA* | ENSG00000100906 | -3.88707 | 4.27E-11 |
| *PCDHA12* | ENSG00000251664 | -8.89581 | 5.35E-11 |
| *SDC1* | ENSG00000115884 | -3.58472 | 9.29E-11 |
| *PCDHB13* | ENSG00000187372 | -10.6909 | 1.89E-10 |
| *MAP10* | ENSG00000212916 | -10.6119 | 2.79E-10 |
| *KRT83* | ENSG00000170523 | -8.418 | 3.58E-10 |
| *MAP2* | ENSG00000078018 | -4.13019 | 3.65E-10 |
| *PCDHB14* | ENSG00000120327 | -6.13142 | 4.48E-10 |
| *ADAMTS19* | ENSG00000145808 | -3.3851 | 5.37E-10 |
| *CCND1* | ENSG00000110092 | -3.27663 | 7.67E-10 |
| *PARP10* | ENSG00000178685 | -4.28497 | 8.90E-10 |
| *NPR3* | ENSG00000113389 | -4.42421 | 1.04E-09 |
| *PCDHB16* | ENSG00000272674 | -10.2591 | 1.70E-09 |
| *MMP17* | ENSG00000198598 | -4.00925 | 3.25E-09 |
| *PCDHA7* | ENSG00000204963 | -10.3054 | 3.99E-09 |
| *CDH13* | ENSG00000140945 | -10.0494 | 4.07E-09 |
| *WNT4* | ENSG00000162552 | -5.39344 | 6.65E-09 |
| *SRMS* | ENSG00000125508 | -6.82755 | 8.40E-09 |
| *RAB20* | ENSG00000139832 | -3.55444 | 8.65E-09 |
| *PCDHGA1* | ENSG00000204956 | -5.43062 | 8.80E-09 |
| *MACC1* | ENSG00000183742 | -9.69882 | 1.00E-08 |
| *SOX11* | ENSG00000176887 | -9.53339 | 1.01E-08 |
| *CD22* | ENSG00000012124 | -6.04625 | 1.29E-08 |
| *BCL6* | ENSG00000113916 | -3.39678 | 1.40E-08 |
| *NRCAM* | ENSG00000091129 | -3.1041 | 4.12E-08 |
| *COL12A1* | ENSG00000111799 | -3.33952 | 4.12E-08 |
| *TP63* | ENSG00000073282 | -5.98185 | 4.95E-08 |
| *PCDHGB1* | ENSG00000254221 | -5.03612 | 5.86E-08 |
| *ADGRF4* | ENSG00000153294 | -5.60247 | 6.66E-08 |
| *TNFRSF18* | ENSG00000186891 | -9.33523 | 6.80E-08 |
| *RAB37* | ENSG00000172794 | -4.28798 | 7.70E-08 |
| *KIF1A* | ENSG00000130294 | -3.36972 | 9.76E-08 |
| *ARHGEF5* | ENSG00000050327 | -9.3588 | 9.81E-08 |
| *LMNA* | ENSG00000160789 | -3.45521 | 9.81E-08 |
| *CDH24* | ENSG00000139880 | -3.24053 | 1.76E-07 |
| *KRT17* | ENSG00000128422 | -6.0269 | 1.82E-07 |
| *AJAP1* | ENSG00000196581 | -9.22878 | 1.85E-07 |
| *PCDHA13* | ENSG00000239389 | -9.39029 | 2.69E-07 |
| *LAMA5* | ENSG00000130702 | -3.45141 | 2.76E-07 |
| *NRBP1* | ENSG00000115216 | -2.8634 | 3.01E-07 |
| *EMP2* | ENSG00000213853 | -2.6466 | 5.67E-07 |
| *ADGRG6* | ENSG00000112414 | -3.10278 | 5.95E-07 |
| *PCDHGB2* | ENSG00000253910 | -4.95149 | 6.53E-07 |
| *COL18A1* | ENSG00000182871 | -2.97089 | 7.64E-07 |
| *ESPN* | ENSG00000187017 | -10.4701 | 8.41E-07 |
| *MMP15* | ENSG00000102996 | -2.90133 | 1.17E-06 |
| *VASP* | ENSG00000125753 | -2.76056 | 1.20E-06 |
| *MUC1* | ENSG00000185499 | -3.0739 | 1.25E-06 |
| *KRT24* | ENSG00000167916 | -8.8525 | 1.40E-06 |
| *MIR4737* | ENSG00000264049 | -4.26976 | 1.67E-06 |
| *PCDHAC1* | ENSG00000248383 | -8.7053 | 1.92E-06 |
| *CEACAM6* | ENSG00000086548 | -8.32546 | 2.00E-06 |
| *PCDHB9* | ENSG00000177839 | -8.63476 | 2.60E-06 |
| *TFPI* | ENSG00000003436 | -3.14726 | 2.68E-06 |
| *TUBB3* | ENSG00000258947 | -4.21155 | 2.96E-06 |
| *TNFSF10* | ENSG00000121858 | -4.99596 | 3.23E-06 |
| *S100P* | ENSG00000163993 | -9.64223 | 3.28E-06 |
| *JDP2* | ENSG00000140044 | -2.69422 | 3.58E-06 |
| *MAMDC4* | ENSG00000177943 | -2.97366 | 3.67E-06 |
| *PCDHB8* | ENSG00000120322 | -8.09131 | 3.92E-06 |
| *NKILA* | ENSG00000278709 | -2.9481 | 4.27E-06 |
| *HSPA6* | ENSG00000173110 | -4.22098 | 4.52E-06 |
| *MAP3K8* | ENSG00000107968 | -2.97908 | 6.03E-06 |
| *MUC3A* | ENSG00000169894 | -4.26317 | 6.56E-06 |
| *JUND* | ENSG00000130522 | -2.45081 | 8.10E-06 |
| *CAMK2B* | ENSG00000058404 | -3.09354 | 9.21E-06 |
| *PCDHA10* | ENSG00000250120 | -6.38713 | 9.52E-06 |
| *CAV1* | ENSG00000105974 | -2.46421 | 9.64E-06 |
| *ABLIM2* | ENSG00000163995 | -3.21067 | 9.64E-06 |
| *PRKCD* | ENSG00000163932 | -2.65035 | 1.29E-05 |
| *NOTCH3* | ENSG00000074181 | -2.93517 | 1.34E-05 |
| *AKT1* | ENSG00000142208 | -2.73676 | 1.74E-05 |
| *KRT13* | ENSG00000171401 | -8.21214 | 1.74E-05 |
| *PCDHB4* | ENSG00000081818 | -7.64107 | 1.94E-05 |
| *NPDC1* | ENSG00000107281 | -2.44297 | 1.94E-05 |
| *TNFAIP2* | ENSG00000185215 | -2.58357 | 1.98E-05 |
| *NECTIN2* | ENSG00000130202 | -2.58125 | 2.00E-05 |
| *ITGAM* | ENSG00000169896 | -6.14166 | 2.39E-05 |
| *ARPC1B* | ENSG00000130429 | -2.79233 | 2.86E-05 |
| *AREG* | ENSG00000109321 | -3.63775 | 2.99E-05 |
| *FILIP1L* | ENSG00000168386 | -3.23019 | 3.01E-05 |
| *MMP19* | ENSG00000123342 | -7.54883 | 3.03E-05 |
| *MMP9* | ENSG00000100985 | -4.37314 | 3.09E-05 |
| *PTPRJ* | ENSG00000149177 | -2.31114 | 3.13E-05 |
| *FLNA* | ENSG00000196924 | -2.75191 | 3.26E-05 |
| *CAPN1* | ENSG00000014216 | -2.69277 | 3.35E-05 |
| *HSPD1P11* | ENSG00000251348 | -4.13806 | 3.55E-05 |
| *MIR149* | ENSG00000207611 | -4.66217 | 3.89E-05 |
| *KISS1* | ENSG00000170498 | -7.93301 | 4.80E-05 |
| *MAPKAPK2* | ENSG00000162889 | -2.35947 | 4.95E-05 |
| *PXN* | ENSG00000089159 | -2.27782 | 4.97E-05 |
| *VDR* | ENSG00000111424 | -2.39643 | 5.42E-05 |
| *SMAGP* | ENSG00000170545 | -2.34217 | 5.44E-05 |
| *CD63* | ENSG00000135404 | -2.34269 | 5.45E-05 |
| *RAB15* | ENSG00000139998 | -2.26252 | 7.38E-05 |
| *CARD9* | ENSG00000187796 | -2.66765 | 7.40E-05 |
| *PCDHGA5* | ENSG00000253485 | -7.29152 | 7.63E-05 |
| *BBC3* | ENSG00000105327 | -2.52634 | 7.68E-05 |
| *TNS2* | ENSG00000111077 | -2.59524 | 7.97E-05 |
| *CEACAM5* | ENSG00000105388 | -7.2521 | 8.32E-05 |
| *KRT16* | ENSG00000186832 | -7.79991 | 9.45E-05 |
