# Supplementary material for: A single cysteine residue in vimentin regulates long non-coding RNA XIST to suppress epithelial–mesenchymal transition and stemness in breast cancer
Source: eLife. 2025 Jul 21;14:RP104191. doi: 10.7554/eLife.104191 (PMC12279371; doi:10.7554/eLife.104191)
Supplement: Supplementary file 3. [file elife-104191-supp3.docx]

**Supplementary File 3: List of upregulated lnRNAs (cut off padj=0.00009).**

| **Gene Name** | **Gene ID** | **Log2fold Change** | **padj** |
| --- | --- | --- | --- |
| *XIST* | ENSG00000229807 | 11.79645137 | 1.96E-93 |
| *LINC02381* | ENSG00000250742 | 6.953445703 | 1.78E-19 |
| *PURPL* | ENSG00000250337 | 6.328863193 | 2.34E-19 |
| *MIR222HG* | ENSG00000270069 | 5.994059507 | 1.56E-14 |
| *AC027031.2* | ENSG00000254615 | 5.128079486 | 9.98E-13 |
| *AL035446.1* | ENSG00000234147 | 7.509471414 | 8.27E-12 |
| *AL133325.3* | ENSG00000278041 | 10.25216513 | 1.60E-10 |
| *FAM239B* | ENSG00000205663 | 9.696877626 | 1.26E-08 |
| *LINC02241* | ENSG00000251629 | 9.620849091 | 1.76E-08 |
| *AC103702.2* | ENSG00000272763 | 9.584780353 | 2.07E-08 |
| *TSIX* | ENSG00000270641 | 8.852012634 | 6.88E-08 |
| *AC015522.1* | ENSG00000254202 | 9.251935664 | 8.91E-08 |
| *LINC00491* | ENSG00000250682 | 9.186133789 | 1.18E-07 |
| *RAMP2-AS1* | ENSG00000197291 | 6.877939113 | 1.77E-07 |
| *LINC01033* | ENSG00000249069 | 8.819571635 | 5.80E-07 |
| *AL356489.2* | ENSG00000260947 | 8.750532639 | 7.92E-07 |
| *AL513318.2* | ENSG00000269994 | 8.756358592 | 8.03E-07 |
| *SOX21-AS1* | ENSG00000227640 | 6.630286843 | 1.45E-06 |
| *PINCR* | ENSG00000224294 | 8.499738862 | 2.31E-06 |
| *LINC00470* | ENSG00000132204 | 6.394021607 | 3.86E-06 |
| *AL445250.1* | ENSG00000225096 | 7.810485755 | 4.19E-06 |
| *LINC01234* | ENSG00000249550 | 7.756791857 | 5.14E-06 |
| *AC233976.1* | ENSG00000229151 | 8.283465103 | 5.28E-06 |
| *SH3RF3-AS1* | ENSG00000259863 | 8.175078669 | 8.17E-06 |
| *FOXCUT* | ENSG00000280916 | 6.149454375 | 9.33E-06 |
| *DSCR8* | ENSG00000198054 | 8.096367971 | 1.08E-05 |
| *AL158055.1* | ENSG00000226530 | 7.990362422 | 1.63E-05 |
| *AC025575.2* | ENSG00000258053 | 7.99019662 | 1.73E-05 |
| *FENDRR* | ENSG00000268388 | 7.880557066 | 2.52E-05 |
| *ERVMER61-1* | ENSG00000230426 | 7.327968096 | 2.59E-05 |
| *ZNF582-AS1* | ENSG00000267454 | 7.854726374 | 2.92E-05 |
| *LINC02315* | ENSG00000251363 | 7.830396631 | 3.02E-05 |
| *Z68871.1* | ENSG00000239407 | 2.943656954 | 3.07E-05 |
| *AC009275.1* | ENSG00000273297 | 4.707067687 | 3.53E-05 |
| *LINC01976* | ENSG00000261514 | 7.788306456 | 3.58E-05 |
| *LINC00896* | ENSG00000236499 | 5.2395894 | 3.58E-05 |
| *AL365361.1* | ENSG00000259834 | 7.769445937 | 3.82E-05 |
| *LINC00648* | ENSG00000259129 | 4.74170971 | 4.18E-05 |
| *AC027020.2* | ENSG00000270127 | 3.530275396 | 4.44E-05 |
| *AC021504.1* | ENSG00000267313 | 7.675041377 | 5.50E-05 |
| *AL022324.3* | ENSG00000272942 | 7.630929262 | 6.47E-05 |
| *LINC01297* | ENSG00000274827 | 7.653421816 | 6.76E-05 |
| *LINC01876* | ENSG00000226383 | 4.271039981 | 7.16E-05 |
| *AL592295.4* | ENSG00000283696 | 5.060160122 | 8.60E-05 |
