# Supplementary material for: A single cysteine residue in vimentin regulates long non-coding RNA XIST to suppress epithelial–mesenchymal transition and stemness in breast cancer
Source: eLife. 2025 Jul 21;14:RP104191. doi: 10.7554/eLife.104191 (PMC12279371; doi:10.7554/eLife.104191)
Supplement: Supplementary file 4. [file elife-104191-supp4.docx]

**Supplementary File 4: List of downregulated lnRNAs (cut off padj=0.00009).**

| **Gene name** | **Gene ID** | **log2Fold change** | **padj** |
| --- | --- | --- | --- |
| *GATA3-AS1* | ENSG00000197308 | -8.40024 | 1.71E-29 |
| *MIR9-3HG* | ENSG00000255571 | -7.74173 | 2.47E-17 |
| *AP001816.1* | ENSG00000254531 | -5.80986 | 4.42E-17 |
| *C17orf82* | ENSG00000187013 | -6.65662 | 1.33E-16 |
| *AC144831.1* | ENSG00000261888 | -9.6419 | 9.19E-15 |
| *LINC01293* | ENSG00000230836 | -7.1369 | 1.26E-14 |
| *LINC00992* | ENSG00000248663 | -4.72382 | 3.88E-14 |
| *AC004233.3* | ENSG00000272079 | -5.15436 | 6.35E-12 |
| *AC074135.1* | ENSG00000267886 | -11.218 | 1.73E-11 |
| *AC020916.1* | ENSG00000267519 | -4.66826 | 5.24E-11 |
| *AC141928.1* | ENSG00000250986 | -4.7547 | 1.02E-10 |
| *LINC01468* | ENSG00000231131 | -10.7134 | 2.45E-10 |
| *AL355001.2* | ENSG00000275964 | -3.68048 | 2.62E-10 |
| *AL135818.2* | ENSG00000260810 | -6.20619 | 1.13E-09 |
| *AL035661.1* | ENSG00000274173 | -10.5793 | 1.21E-09 |
| *AC044784.1* | ENSG00000223808 | -10.2426 | 1.94E-09 |
| *FAM225A* | ENSG00000231528 | -10.1514 | 3.87E-09 |
| *AC144450.1* | ENSG00000203635 | -10.0804 | 4.29E-09 |
| *LINC00052* | ENSG00000259527 | -9.73082 | 6.00E-09 |
| *PSMG3-AS1* | ENSG00000230487 | -3.6883 | 6.33E-09 |
| *AC015712.1* | ENSG00000232386 | -6.69834 | 7.38E-09 |
| *AL590004.4* | ENSG00000260604 | -9.9135 | 8.83E-09 |
| *AP000439.2* | ENSG00000255774 | -9.92916 | 9.10E-09 |
| *LINC01503* | ENSG00000233901 | -4.62263 | 1.93E-08 |
| *AC096733.2* | ENSG00000273472 | -3.53195 | 2.25E-08 |
| *AC022034.2* | ENSG00000237807 | -9.66219 | 2.45E-08 |
| *C9orf163* | ENSG00000196366 | -4.3994 | 3.78E-08 |
| *AL365181.2* | ENSG00000272068 | -7.28242 | 4.12E-08 |
| *PVT1* | ENSG00000249859 | -3.06311 | 5.25E-08 |
| *AL390719.2* | ENSG00000272141 | -9.58839 | 5.33E-08 |
| *C9orf106* | ENSG00000179082 | -9.43835 | 6.93E-08 |
| *AC015922.3* | ENSG00000265519 | -9.3775 | 9.11E-08 |
| *FAM111A-DT* | ENSG00000245571 | -9.37063 | 9.83E-08 |
| *LINC00886* | ENSG00000240875 | -4.26358 | 1.17E-07 |
| *MIR200CHG* | ENSG00000257084 | -9.35895 | 1.27E-07 |
| *AP003559.1* | ENSG00000256443 | -5.32785 | 1.80E-07 |
| *AL512625.2* | ENSG00000229422 | -3.34184 | 1.81E-07 |
| *AC012307.1* | ENSG00000228873 | -9.38298 | 2.10E-07 |
| *AC008014.1* | ENSG00000257261 | -4.13124 | 2.36E-07 |
| *AC009237.14* | ENSG00000272913 | -9.18672 | 2.56E-07 |
| *AC008556.1* | ENSG00000277013 | -6.96466 | 2.76E-07 |
| *SNHG19* | ENSG00000260260 | -2.94687 | 4.38E-07 |
| *LINC02568* | ENSG00000259459 | -9.0107 | 5.33E-07 |
| *AC007342.4* | ENSG00000261804 | -8.64953 | 5.72E-07 |
| *LINC00847* | ENSG00000245060 | -3.02844 | 1.03E-06 |
| *AC010735.2* | ENSG00000272622 | -6.74235 | 1.26E-06 |
| *LINC01213* | ENSG00000244541 | -8.76016 | 1.51E-06 |
| *AC061992.1* | ENSG00000266970 | -4.75367 | 1.94E-06 |
| *DIO3OS* | ENSG00000258498 | -5.96001 | 2.22E-06 |
| *AC006206.2* | ENSG00000256417 | -8.65963 | 2.38E-06 |
| *LINC02021* | ENSG00000249846 | -3.41324 | 2.86E-06 |
| *AC090114.2* | ENSG00000273270 | -2.77024 | 3.05E-06 |
| *AC092279.1* | ENSG00000268362 | -3.31659 | 3.28E-06 |
| *AC096888.1* | ENSG00000244564 | -8.56423 | 3.50E-06 |
| *AL021807.1* | ENSG00000272468 | -4.55678 | 3.82E-06 |
| *FAM225B* | ENSG00000225684 | -8.62311 | 4.18E-06 |
| *EPB41L4A-AS2* | ENSG00000278921 | -3.56267 | 4.90E-06 |
| *SNHG18* | ENSG00000250786 | -7.91721 | 7.49E-06 |
| *LINC01750* | ENSG00000231437 | -6.34424 | 8.06E-06 |
| *AC083967.1* | ENSG00000254337 | -7.87222 | 8.32E-06 |
| *LINC02015* | ENSG00000231574 | -7.86906 | 8.34E-06 |
| *LINC01016* | ENSG00000249346 | -7.82734 | 9.95E-06 |
| *AC100860.1* | ENSG00000253266 | -8.34184 | 1.10E-05 |
| *LINC00885* | ENSG00000224652 | -7.93839 | 1.11E-05 |
| *AL645608.9* | ENSG00000273443 | -8.35171 | 1.13E-05 |
| *HAGLROS* | ENSG00000226363 | -4.28912 | 1.18E-05 |
| *UCA1* | ENSG00000214049 | -8.26012 | 1.23E-05 |
| *AC147651.1* | ENSG00000223855 | -4.18837 | 1.30E-05 |
| *C15orf59-AS1* | ENSG00000260469 | -3.70555 | 1.30E-05 |
| *LINC00628* | ENSG00000280924 | -8.21329 | 1.60E-05 |
| *AL357558.1* | ENSG00000228503 | -8.18389 | 1.89E-05 |
| *AL357558.2* | ENSG00000234967 | -8.13677 | 2.22E-05 |
| *AC005332.6* | ENSG00000277476 | -2.89766 | 2.22E-05 |
| *AC091271.1* | ENSG00000273702 | -2.82113 | 2.35E-05 |
| *AL117329.1* | ENSG00000224271 | -8.08866 | 2.67E-05 |
| *AP000439.5* | ENSG00000285094 | -8.09905 | 2.97E-05 |
| *LINC00898* | ENSG00000205634 | -8.1746 | 3.47E-05 |
| *LINC01671* | ENSG00000225431 | -7.96645 | 4.27E-05 |
| *AC011416.3* | ENSG00000283897 | -7.94267 | 4.61E-05 |
| *LINC00994* | ENSG00000189196 | -7.95062 | 4.62E-05 |
| *LINC00865* | ENSG00000232229 | -7.4451 | 4.71E-05 |
| *AP002884.1* | ENSG00000250303 | -3.14553 | 4.71E-05 |
| *AC005618.1* | ENSG00000272070 | -4.40236 | 4.81E-05 |
| *LINC01376* | ENSG00000236204 | -4.25144 | 5.51E-05 |
| *AL133342.1* | ENSG00000278231 | -7.89219 | 5.58E-05 |
| *ERVE-1* | ENSG00000267259 | -7.36674 | 5.74E-05 |
| *AL356740.1* | ENSG00000267868 | -5.99969 | 6.19E-05 |
| *LINC01918* | ENSG00000226508 | -4.15419 | 6.49E-05 |
| *BX539320.1* | ENSG00000278869 | -7.88744 | 7.00E-05 |
| *LINC01665* | ENSG00000235343 | -7.82432 | 7.68E-05 |
| *AC005993.1* | ENSG00000266869 | -7.2285 | 8.47E-05 |
| *LINC01637* | ENSG00000237476 | -5.0912 | 8.73E-05 |
| *LINC01977* | ENSG00000262772 | -3.07166 | 9.91E-05 |
