# Supplementary material for: A single cysteine residue in vimentin regulates long non-coding RNA XIST to suppress epithelial–mesenchymal transition and stemness in breast cancer
Source: eLife. 2025 Jul 21;14:RP104191. doi: 10.7554/eLife.104191 (PMC12279371; doi:10.7554/eLife.104191)
Supplement: Supplementary file 5. [file elife-104191-supp5.docx]

**Supplementary File 5: List of primers used for qPCR**

| Gene | Product Size (bp) | Primer Sequence  5`-3` | NM # |
| --- | --- | --- | --- |
| *VIM* | 123 | F>AGGTGGACCAGCTAACCAAC | NM_003380 |
|  |  | R>TTTCGGCTTCCTCTCTCTGA |  |
| *YAP1* | 83 | F>CCCAGATGAACGTCACAGC | NM_006106 |
|  |  | R>GATTCTCTGGTTCATGGCTGA |  |
| *POLR2A* | 73 | F>GCAAATTCACCAAGAGAGAC | NM_000937 |
|  |  | R>CACGTCGACAGGAACATCAG |  |
| *KRT18* | 117 | F>TGATGACACCAATATCACACGA | NM_000224 |
|  |  | R>ATCTGGGCTTGTAGGCCTTT |  |
| *KRT19* | 126 | F>GCCACTACTACACGACCATCC | NM_002276 |
|  |  | R>CAAACTTGGTTCGGAAGTCAT |  |
| *KRT80* | 257 | F> AGGATGCCAAGACCAAGCTG | NM_001081492 |
|  |  | R>CAGCTACAGGGAACATAAGGGG |  |
| *SNAI*1 | 92 | F>TACAGCGAGCTGCAGGACT | NM_005985 |
|  |  | R>ATCTCCGGAGGTGGGATG |  |
| *SNAI2* | 984 | F>TGGTTGCTTCAAGGACACAT | NM_003068.4 |
|  |  | R>GCAAATGCTCTGTTGCAGTG |  |
| *TWIST1* | 124 | F>AGCTACGCCTTCTCGGTCT | NM_000474 |
|  |  | R>CCTTCTCTGGAAACAATGACATC |  |
| *ZEB1* | 74 | F>TGCAGTTTTCAAAGTTAGGAACAA | NM_001128128 |
|  |  | R>TGTTGCTCTCTGAGTCATTAAGGT |  |
| *ZEB2* | 86 | F>TTGCTCCAAGATGTGTGAGG | NM_001171653 |
|  |  | R>TGTGGGGCTCCAGATATACAC |  |
| *KRT8* | 282 | F>AGCTTCTCCGCTCCTTCTAGG | NM_002273 |
|  |  | R>CAGGCTCTGGTTGACCGTAA |  |
| *XIST* | 183 | F>AGATCTTCCTCAGAAGAATAGG | NR_001564.2 |
|  |  | R>TTTATCTTCCTATCTGGGACC |  |
