# Supplementary material for: A single cysteine residue in vimentin regulates long non-coding RNA XIST to suppress epithelial–mesenchymal transition and stemness in breast cancer
Source: eLife. 2025 Jul 21;14:RP104191. doi: 10.7554/eLife.104191 (PMC12279371; doi:10.7554/eLife.104191)
Supplement: Supplementary file 6. [file elife-104191-supp6.docx]

**Supplementary File 6: List of primary and secondary antibodies used in this research work**

| Antibody | Dilution | Host | Catalogue # | Supplier |
| --- | --- | --- | --- | --- |
| Anti-vimentin V9 | IF=1:700 | Mouse | ab8069 | Abcam, UK |
|  | WB=1:2000 |  |  |  |
| Anti-cytokeratin K8 | IF=1:100 | Rabbit | ab53280 | Abcam, UK |
|  | WB=1:500 |  |  |  |
| Anti-cytokeratin K18 | IF=1:500 | Rabbit | ab24561 | Abcam, UK |
|  | WB=1:1000 |  |  |  |
| Anti-CDH2 | WB= 1:500 | Mouse | CA1029 | Merck Millipore, UK |
| Anti-Twist1 | WB= 1:500 | Rabbit | PA5-86070 | Thermo Fisher Scientific, UK |
| Anti-GAPDH | WB= 1:2000 | Rabbit | Ab9485 | Abcam, UK |
| Phalloidin, Alexa Flour568 conjugated | IF= 1:400 | N/A | A12380 | Life Technologies, UK |
| Anti-mouse Alexa Fluor® 488 IgG H+L | IF=1:1000 | Goat | A-11001 | Life Technologies, UK |
| Anti-mouse Alexa Fluor® 594 IgG H+L | IF=1:1000 | Goat | A-11005 | Molecular Probes, UK |
| Anti-rabbit Alexa Fluor®488 IgG H+L | IF=1:1000 | Goat | A-11008 | Life Technologies, UK |
| Anti-rabbit Alexa Fluor®594 IgG H+L | IF=1:1000 | Goat | A-11012 | Life Technologies, UK |
| Mouse IgG peroxidase conjugated | WB= 1:1000 | Goat | NA931V | GE Healthcare, UK |
| Rabbit IgG peroxidase conjugated | WB=1:1000 | Donkey | NA934V | GE Healthcare, UK |
| BD Pharmingen™ APC Rat Anti-Human CD201 | 1:20 | Rat | 563622 | BD Pharmingen™ |
| BD Horizon™ RY586 Mouse Anti-Human CD56 | 1:20 | Mouse | 568150 | BD Pharmingen™ |
| BD Horizon™ Fixable Viability Stain 575V | 1:1000 |  | 565694 | BD Pharmingen™ |
| Rabbit anti-NCAM-1/CD56 antibody (clone E7X9M) | 1:400 | Rabbit | 99746 | Cell Signaling Technology, Danvers, MA |
| Goat anti-CD201 | 10μg/ml | Goat | AF2245 | R&D Systems, Minneapolis, MN |
