# Supplementary material for: A single cysteine residue in vimentin regulates long non-coding RNA XIST to suppress epithelial–mesenchymal transition and stemness in breast cancer
Source: eLife. 2025 Jul 21;14:RP104191. doi: 10.7554/eLife.104191 (PMC12279371; doi:10.7554/eLife.104191)
Supplement: Supplementary file 7. [file elife-104191-supp7.docx]

**Supplementary File 7: List of primers used for making *XIST* shRNA constructs and site directed mutagenesis at C328 and Y117.**

| *XIST*_shRNA1 | F:5'GATCTGGAATATTTGCAATTATAAtacctgacccataTTATAATTGCAAATATTCCTTTTTC3'  R:5'TCGAGAAAAAGGAATATTTGCAATTATAAtatgggtcaggtaTTATAATTGCAAATATTCCA3' |
| --- | --- |
| *XIST*_shRNA2 | F:5'GATCTGGATATATTGCTTAATTTAtacctgacccataTAAATTAAGCAATATATCCTTTTTC3'  R:5'TCGAGAAAAAGGATATATTGCTTAATTTAtatgggtcaggtaTAAATTAAGCAATATATCCA3' |
| *XIST*_shRNA3 | F:5'GATCTGAATATTTGCAATTATATAtacctgacccataTATATAATTGCAAATATTCTTTTTC3'  R:5'TCGAGAAAAAGAATATTTGCAATTATATAtatgggtcaggtaTATATAATTGCAAATATTCA3' |
| *XIST*_shRNA4 | F:5'GATCTGCTTTAATTACATTTAATAtacctgacccataTATTAAATGTAATTAAAGCTTTTTC3'  R:5'TCGAGAAAAAGCTTTAATTACATTTAATAtatgggtcaggtaTATTAAATGTAATTAAAGCA3' |
| C328S-VIM | F:5’CAGGTGCAGTCCCTCACCTCTGAAGTGGATGCCCTTAAA3’  R:5’TTTAAGGGCATCCACTTCAGAGGTGAGGGACTGCACCT3’ |
| Y117L-VIM | F:5’AATGACCGCTTCGCCAACCTCATCGACAAGGTGCGCTT3’  R:5’AAGCGCACCTTGTCGATGAGGTTGGCGAAGCGGTCATT3’ |
| The lower case sequence in shRNA constructs forms the loop containing 13 nucleotides before the dicer acts to produce the mature shRNA. | |
